# A Robust 3D-Based Color Correction Approach for Texture Mapping Applications

**DOI:** 10.3390/s22051730

**Published:** 2022-02-23

**Authors:** Daniel Coelho, Lucas Dal’Col, Tiago Madeira, Paulo Dias, Miguel Oliveira

**Affiliations:** 1 Department of Mechanical Engineering, University of Aveiro, 3810-193 Aveiro, Portugal; lucasrdalcol@ua.pt (L.D.); mriem@ua.pt (M.O.); 2 Institute of Electronics and Informatics Engineering of Aveiro, University of Aveiro, 3810-193 Aveiro, Portugal; tiagomadeira@ua.pt (T.M.); paulo.dias@ua.pt (P.D.); 3 Department of Electronics, Telecommunications and Informatics, University of Aveiro, 3810-193 Aveiro, Portugal

**Keywords:** color correction, texture mapping, joint image histogram, color mapping function

## Abstract

Texture mapping of 3D models using multiple images often results in textured meshes with unappealing visual artifacts known as texture seams. These artifacts can be more or less visible, depending on the color similarity between the used images. The main goal of this work is to produce textured meshes free of texture seams through a process of color correcting all images of the scene. To accomplish this goal, we propose two contributions to the state-of-the-art of color correction: a pairwise-based methodology, capable of color correcting multiple images from the same scene; the application of 3D information from the scene, namely meshes and point clouds, to build a filtering procedure, in order to produce a more reliable spatial registration between images, thereby increasing the robustness of the color correction procedure. We also present a texture mapping pipeline that receives uncorrected images, an untextured mesh, and point clouds as inputs, producing a final textured mesh and color corrected images as output. Results include a comparison with four other color correction approaches. These show that the proposed approach outperforms all others, both in qualitative and quantitative metrics. The proposed approach enhances the visual quality of textured meshes by eliminating most of the texture seams.

## 1. Introduction

The creation of 3D models from the captured shape and appearance of objects is known as 3D reconstruction. It is a widely researched topic in areas such as computer graphics [1] and computer vision [2], and has recently gained attention in others, namely robotics [3], autonomous driving [4], medical applications [5], cultural heritage [6] and agriculture [7].

Texture mapping is the colorization of a 3D mesh using a single image [8,9]. However, in more recent applications, texture mapping has been applied to cases where several overlapping images are available to colorize the mesh [10,11]. The overlap will often occur in irregular regions, which creates an intricate combination of dependencies. This entails the existence of a mechanism to handle the redundant photometric information, and is often referred to as multi-view texture mapping [12,13,14,15]. The problem is generally solved in two different ways: by selecting a single image from the set of possible images, or by fusing the texture from all available images. Several authors have proposed methodologies to carry out the fusion, based on different forms of weighted average of the contributions of textures in the image space [16,17]. However, these approaches are highly sensitive to inaccuracies in camera pose estimation, as even slight misalignments may generate ghost and blurring artifacts in the textures, which are not visually appealing [18]. Still, selecting a single image to be used for texture mapping raises the problem of choosing the most adequate one from a discrete set of possibilities and additionally, how to produce a consistent selection of images for all the faces in the 3D mesh. Some authors propose to employ a Markov random field to select the images [19]. Others have used graph optimization mechanisms [20], or optimization procedures that minimize discontinuities between neighbouring faces [21]. These approaches address the problem of blurring and ghost artifacts. However, particularly in regions where the borders of the selected images meet, visual seam artifacts are often noticeable. The seams are more or less visible depending on the similarity of colors in the images, which is why a proper color correction of the images is crucial to achieve seamless texture mapping. This may be carried out using a form of post-processing operation. For example, in [22] authors propose to use Poisson image editing techniques to smooth the texture. Color correction can be defined as the general problem of compensating the photometrical disparities between two coarsely geometrically registered images. In other words, color correction consists of transferring the color palette of a reference image, usually called source image (**S**), to a target image (**T**) [23].

There are several color correction approaches in the literature, however, the majority of them focus on correcting a pair of images, whereas our objective is to increase the level of similarity of multiple images. Color correction approaches, in a general sense, can be divided into two main classes: model-based parametric and model-less non-parametric.

Model-based parametric approaches assume that the color distribution of the images follows some statistical distribution. In 2001, Reinhard et al. [24] used simple Gaussians to model the global color distributions of images **S** and **T**, proposing a linear transformation that relates the mean and the standard deviation of both images in the lαβ color space. This work was one of the first model-based parametric methods for color correction, and is commonly used as baseline method for comparing other color correction approaches [25,26,27]. Additional research focused on improving the modeling of the color distributions of the images, due to the simplicity of Gaussian distributions. For instance, in [28], the authors divided the images into regions, then matched the regions of both images, and for each match of regions applied a similar method to the one proposed in [24]. In addition, the authors also proposed the usage of Gaussian Mixture Models (GMMs) and the Expectation Maximization (EM) algorithm to model the color distribution more accurately. In [29], the authors first divided the images into spatially connected regions using the mean shift algorithm, then each region was modeled as a collection of truncated Gaussians using a maximum likelihood estimation procedure, in order to model more accurately the local color distributions. In [26], the authors proposed an extension of [24] to the RGB color space, by the development of a simple statistic-based method that uses both the mean and covariance matrix. Another problem identified by [24] was the appearance of cross channel artifacts caused by the modelling and correcting of each channel of the images independently. In [27], the authors tried to solve this problem by using 3D GMMs to model the color distribution across the 3 channels of the images. Other model-based parametric approaches use gain compensation techniques [30,31,32], only operating in the intensity channel rather than in the full color space.

Model-less non-parametric approaches make no assumptions about the nature of the color distributions of the images. Usually, in these approaches, a 2D Joint Image Histogram (JIH) is computed from the overlapped areas of two images [23]. The JIH is then used to estimate a Color Mapping Function (CMF). In this process, there are two points that deserve to be highlighted: (a) robust methods are usually required in order to deal with the outliers caused by different illumination, camera exposure variation [33], vignetting effect [34], reflection properties of certain objects, occlusions, capturing angles, among others [23]; (b) the monotonicity property of the CMF has to be maintained [35]. There are several examples of model-less non-parametric approaches, and what differentiates most of the approaches is how they tackle those two. For example, in [35], the authors propose a robust estimation of the Brightness Transfer Function (BTF) from the JIH of two overlapped images. The authors also used dynamic programming to enforce the monotonicity constraint in the estimation of the BTF. In [36,37], the authors solved these two issues in a separated way: first they used a 2D tensor voting to remove the noise and produce an initial estimation of the CMF; then they used a heuristic local adjustment method to regulate the CMF estimation and enforce the monotonicity increasing constraint. The same authors, in [38], experimented the usage of a Bayesian framework to estimate the CMF. In [39], the authors proposed an energy minimization method in the histogram to estimate a robust CMF, while in [40], high-dimensional Bezier patches were used to estimate the CMF. In [41], the authors employed the moving least squares framework with spatial constrains to correct images in the RGB color space. In [33], the authors proposed the usage of a root-polynomial regression that is invariant to the camera exposure variation and scene irradiance. Another option, as seen in [42], is to use the Vandermonde matrix to find an interpolating polynomial.

Color correction approaches can also be divided based on whether they are global or local. Global color correction approaches [24,26,43,44] assume that estimating a single color mapping function for the entire image should be sufficient to perform a proper color correction. In complex scenes, global color correction approaches are often unable to accurately model the color distribution in the images due to factors such as color clusters, differing optics, sensor characteristics, hardware processing employed by video cameras, among others [45]. Local color correction approaches [28,29,35,46,47] go further by segmenting the image into spatially connected regions or color clusters, and then fitting a color mapping function to each of the obtained segments. These approaches are able to model the color distribution more precisely, and, in most cases, this translates to a more accurate color correction process. However, local approaches usually do not allow a direct mapping of color between pixels for a pair of images because they are limited by the accuracy of the geometric registration. As a result, there is either a maximum number or a minimum area of segmented regions for the image [29]. In other words, for very small regions or, in the degenerate case, single pixel regions, the color correction will fail because of misalignment in the region mappings, caused by error in the geometric registration. Statistical inference over small sample sizes is also noisy and there are local color correction problems such as image clarity reduction and structural inconsistency. To tackle these issues [48], proposed the optimization of a residual image between the target image and the corrected one. There are also authors, such as [37,49], who have tried to combine benefits of both approaches by taking into consideration global and local color information.

The two most complete performance evaluations of color correction approaches were carried out in [23] and in [29]. In [23], the authors tested nine color correction algorithms applied to 70 pairs of images. The conclusions of that evaluation state that, in the model-based parametric approaches, gain compensation [32] and local color transfer [28] were the approaches that yielded the best results, when considering the color transfer effectiveness metric. In the model-less non-parametric approaches, the best approaches, considering that same evaluation metric, were the tensor voting in joint image space [37] and brightness transfer function [35]. Furthermore, the authors claim that, using more evaluation metrics, such as extendability, stability, and speed, both the model-based parametric approaches cited above [28,32] achieved the best results. In [29], the authors carried out a performance evaluation of 11 different color correction algorithms, applied to 63 pairs of images and used three evaluation metrics. They demonstrated that their approach, modeling of regions using truncated Gaussians, outperforms all other ten approaches considering the PSNR (peak signal-to-noise ratio) and S-CIELAB (spatial CIELAB) metrics. Furthermore, the authors show that, with respect to color correction performances, the RGB and lαβ color spaces achieve similar results.

The previous lines have detailed several approaches to cope with the problem of color correcting a pair of images. Texture mapping applications often have several images of the scene, and this is one of the shortcomings of the state-of-the-art: most approaches are designed to operate using only two images, and it is not straightforward how those methods could be expanded to accommodate multiple images.

Another relevant issue is the usage of spatial information to enhance the color correction. There are several global approaches [24,26,28], which do not use spatial information as an input to the color correction procedure. However, since they do not require spatial registration between the images, their effectiveness is limited. Some other proposed approaches make use of spatial information, but only at the image level, meaning that they require the images to be geometrically registered with respect to each other. As seen in [23,35], and more recently [29], the usage of this additional information enhances the effectiveness of the color correction with respect to that of global approaches. However, there is additional information that could further enhance the color correction procedure, which has not been used thus far. We refer to the spatial registration, not only between the images in the scene, but also with respect to the range measurements of that scene (the point clouds) and the mesh that represents the surfaces of the scene. Using this data, it is possible to detect occlusions and other situations that may lead to poor performance of the color correction algorithm.

This paper proposes a novel method that attempts to make use of that additional information in order to improve the effectiveness of the color correction procedure. Furthermore, we proposed a pairwise-based methodology that is able to tackle the problem of color correcting multiple images. The procedure works by color correcting all images in the dataset against a common source image, referred to as reference image. We have also implemented two distinct image selection algorithms to assess the performance of the proposed color correction algorithms under the application of different camera selection criteria.

The remainder of the paper is structured as follows: in Section 2, the proposed approach is presented, Section 3 discusses the results, and Section 4 presents the conclusions.

## 2. Proposed Approach

**Figure 1:**
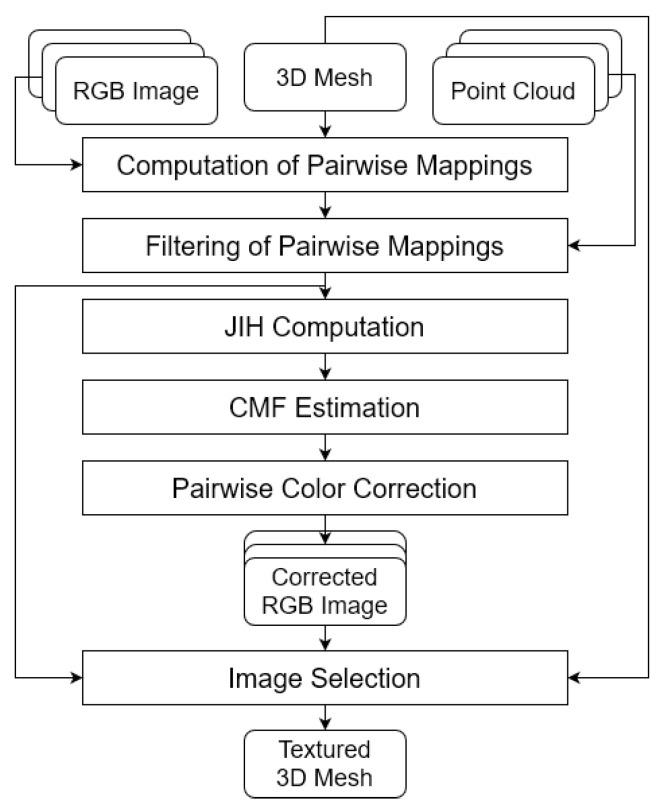
Architecture of the proposed approach. As input, our system receives RGB images, a 3D mesh and registered point clouds from different scans. Firstly, the faces present in the 3D mesh are projected onto the images to create the pairwise mappings. Then, the pairwise mappings are filtered using several filtering techniques. The filtered pairwise mappings are then used to compute a JIH for each pair of images. Subsequently, each JIH is used to estimate a CMF. To produce the corrected RGB images, a pairwise color correction procedure is carried out using the CMFs. At the end, we compute a textured 3D mesh using an image selection technique that receives the corrected RGB images, the 3D mesh and the filtered pairwise mappings.

The architecture of the proposed approach is depicted in Figure 1. As input, our system takes RGB images, registered point clouds from different scans, and a 3D mesh obtained from those point clouds. All RGB images must be geometrically registered with respect to the 3D model, given that our main objective is to use 3D information to enhance the similarity between the images. Firstly, the faces present in the 3D mesh are projected onto the images, to create what we call pairwise mappings, that is, for each of the mesh triangles, we compute the pixel coordinates of the projection corresponding to the three vertices in each pair of images. Then, we apply several techniques to filter the noisy pairwise mappings that would undermine both the color correction process and the texture mapping. After a successful removal of the noisy pairwise mappings, we compute a Joint Image Histogram (JIH) for every pair of images and estimate a Color Mapping Function (CMF) that best fits the JIH data. To finish the color correction procedure, we perform pairwise color correction using the CMFs created in the previous step, effectively correcting all images with respect to a reference image that must be selected by the user. Section 2.5 will provide details on how this selection is carried out. At the end of this step, we produce the corrected RGB images. To analyze the influence of this 3D-based color correction in 3D meshes, we use the corrected images to colorize each face of the 3D mesh using 2 different techniques that will be explained later on. Besides using color corrected images to colorize the 3D mesh, we also make use of the information from the pairwise mappings filtering component to increase the robustness of the image selection technique. At the end, we produce the textured mesh.

**Figure 2:**
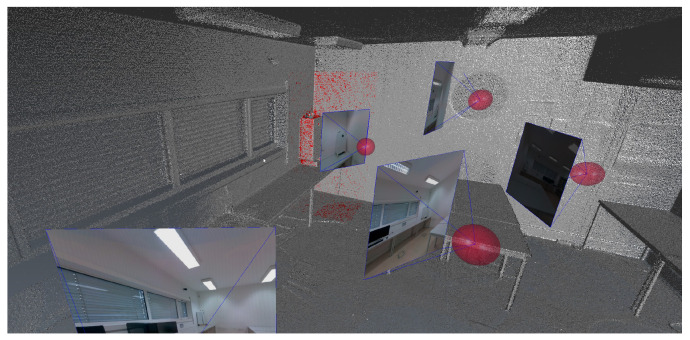

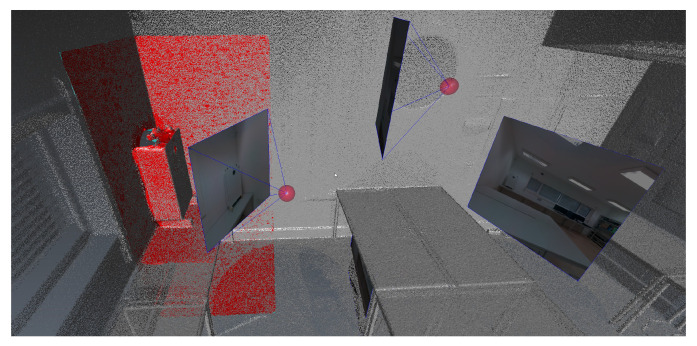
Two viewpoints from the dataset. The gray points represent the point cloud, the red surface represents the 3D mesh, and the red spheres represent the scan locations. The images are also shown, with frustum in blue.

In the remainder of this section, we describe every component of the architecture and present the intermediate results for each of the components. The dataset (Figure 2) used to produce the intermediate results was taken from a laboratory, containing 7 images, 9 point clouds from different scans of the entire room, and a 3D mesh with 46,627 faces and 27,454 vertices from a corner of the room.

### 2.1. Computation of Pairwise Mappings

The first step of the 3D-based color correction is the projection of the mesh faces onto the images. For this purpose, we use the pinhole camera model [50] to compute the projections of all vertices of the faces. The projection of the 3D coordinates ($x_{f,v}\in R^{3}$) of the *v*-th vertex of the *f*-th face, of the set of faces F, onto the *i*-th image ($x_{f,v}^{\left(i\right)}\in Z^{2}$), of the set of images I, can be described as:

(1) $$\begin{array}{cc} \; & x_{f,v}^{\left(i\right)}=K\cdot T_{i}\cdot x_{f,v}\;,\\ \; & \forall f\in F\wedge \forall v\in \{1,2,3\}\wedge \forall i\in I\;, \end{array}$$

where $T_{i}$ represents the extrinsic parameters associated with the camera that took the *i*-th image (3 × 4 matrix) and K represents the camera’s intrinsic parameters (3 × 3 matrix). Since all images were taken using the same camera, K will be considered the same for all images. Throughout the document, we use the ${\left[\cdot \right]}^{\left(i\right)}$ notation to denote a projection onto the *i*-th image. It is important to note that, although we are projecting the vertices of the mesh onto the images, our atomic units are faces and not vertices. Thus, to assess the validity of the projection of the *f*-thx face onto the *i*-th image, two conditions must be fulfilled: all vertices of the *f*-th face must be projected inside the *i*-th image and the *z* component of the 3D coordinates of the *f*-th face vertices, transformed to the camera’s coordinate reference system, must be greater than 0. This assessment can be formulated as:

(2) $$\begin{array}{cc} F^{\left(i\right)}=\{f\in F:0\leq {\left[x_{f,v}^{\left(i\right)}\right]}_{x}\;<W\wedge 0\leq {\left[x_{f,v}^{\left(i\right)}\right]}_{y}\;<H\;\wedge \; & {\left[T_{i}\cdot x_{f,v}\right]}_{z}>0\}\;,\\ \; & \forall v\in \{1,2,3\}\;, \end{array}$$

where $F^{\left(i\right)}$ is a set that contains the faces with valid projection to the *i*-th image, W,H represents the width and height of the image, ${\left[\cdot \right]}_{x}$, ${\left[\cdot \right]}_{y}$, and ${\left[\cdot \right]}_{z}$ are operators that extract the *x*, *y*, and *z* coordinates, respectively.

**Figure 3:**
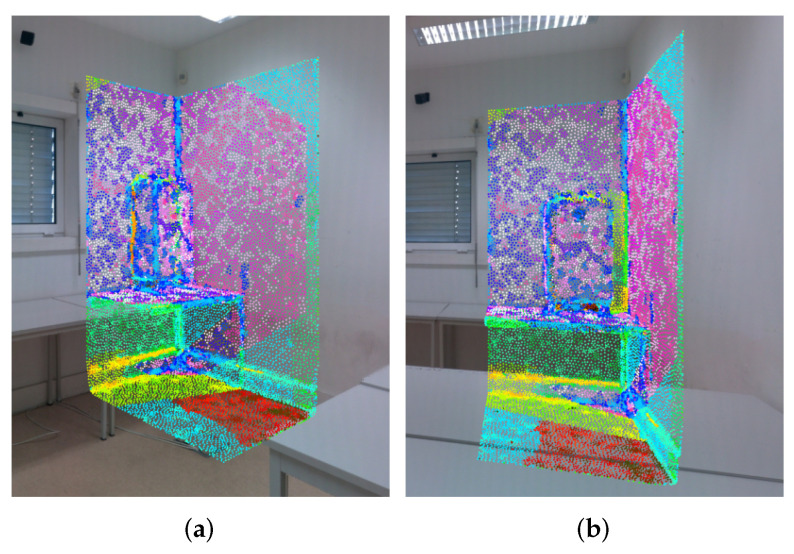
Pairwise mappings between image (**a**) and image (**b**). Points are colored to identify the mappings between images. For instance, in both images, the points in pink belong to the wall.

After the calculation of the valid projections, we compute what we refer to as pairwise mappings. Pairwise mappings are pixel coordinates from a pair of images that correspond to the same projected vertex. Firstly, we loop through each face for every pair of images, and evaluate if the projection of that face is valid, considering both images. Then, we create a pairwise mapping for each vertex that consists of a tuple with two elements $\left(\langle x_{f,v}^{\left(i\right)},x_{f,v}^{\left(k\right)}\rangle \right)$: the pixel coordinates of the vertex projection onto the first image and onto the second image. This procedure can be formulated as:

(3) $$M_{\langle i,k\rangle }=\left\{\langle x_{f,v}^{\left(i\right)},x_{f,v}^{\left(k\right)}\rangle :f\in F^{\left(i\right)}\wedge f\in F^{\left(k\right)}\right\},\forall v\in \left\{1,2,3\right\}\;,$$

where $M_{\langle i,k\rangle }$ is the set that contains the pairwise mappings associated with the *i*-th and *k*-th images. In Figure 3, the pairwise mappings are illustrated by using the same color to showcase correspondence mappings between images.

We believe that the usage of pairwise mappings is more accurate and reliable than the information computed based on the overlapping areas of two images, since it contains photometrical information between pixels that correspond to the same projected vertex, instead of containing photometrical information between regions of pixels that may or may not correspond to the same 3D object. However, the state of the pairwise mappings at this stage of the pipeline is far from optimal, because they contain noisy data caused by the problems mentioned in Section 1: different illumination, camera exposure variation, occlusions, reflection properties of certain objects, capturing angles, among others. In the next section, such problems and the proposed solutions for them will be discussed.

### 2.2. Filtering of Pairwise Mappings

The pairwise mappings computed as described in Section 2.1 contain a significant amount of noise, as can be seen in Figure 3. For example, the red colored pixels should correspond to the same projected 3D object, which is untrue because in Figure 3a those pixels are the projection of the ground, while in Figure 3b those pixels are the projection of the table surface. Inaccurate pairwise mappings are caused by two reasons: occluded faces and registration errors. The most common source of noise in our system are faces occluded in at least one of the images. This happens when two conditions are met: (a) the occluding face intersects the line of sight between the camera and the occluded face; (b) the occluding face is closer to the camera. The usage of pairwise mappings with occluded faces introduces incorrect photometrical data into the system, which undermines the color correction procedure. To tackle this problem, we use two filtering methods, as in [18]: z-buffering and depth consistency. Although these filtering methods aim to tackle the same problem, their scope of work is different, as will be explained below. Regarding the registration errors, we are using professional equipment that guarantees a low average registration error, and therefore, we do not propose solutions to reduce it. Still, even though the average registration error may be low, in situations where the camera viewpoint with respect to the face is excessively oblique, i.e, the angle between the focal axis of the camera and the normal vector of the face is near $90^{\circ }$, the error impact on the accuracy of the pairwise mappings is amplified, potentially leading to inaccurate pairwise mappings. For this reason, we propose an additional filtering method, which we call camera viewpoint filtering, that focuses on discarding pairwise mappings of faces that have an excessive oblique angle with respect to the camera focal axis.

**Figure 4:**
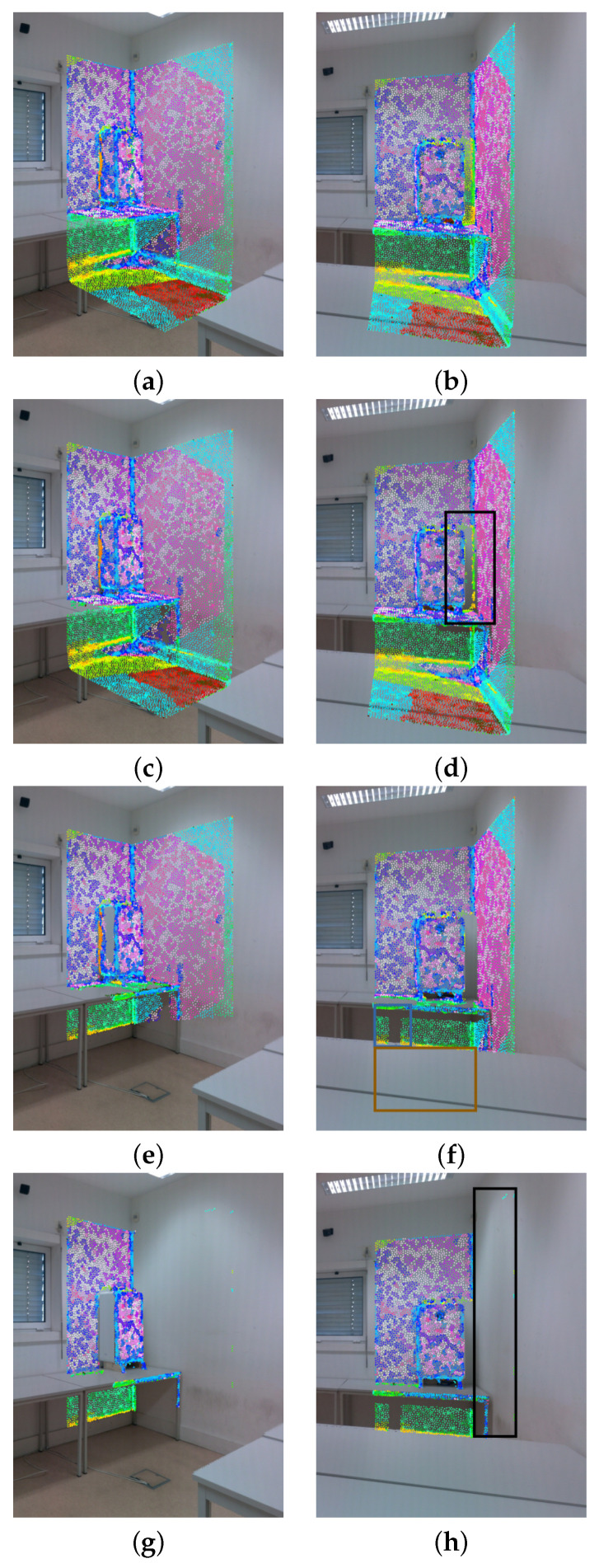
Pairwise mappings filtering procedure: (**a**,**b**) pairwise mappings with no filter applied; (**c**,**d**) pairwise mappings with z-buffering applied; (**e**,**f**) pairwise mappings with z-buffering filter and depth consistency applied; and (**g**,**h**) pairwise mappings with the entire filtering procedure applied.

In order to eliminate the noisy pairwise mappings, we propose a filtering procedure composed of a combination of the three filtering methods described above: z-buffering filtering, depth consistency filtering, and camera viewpoint filtering. Each pairwise mapping computed using Equation (3) is submitted to this procedure, and is only considered valid if it passes the 3 filtering methods. In the next paragraphs, each of the individual falterings will be analyzed in detail, introducing the equations that allow their computation. The evolution of the pairwise mappings throughout the filtering procedure is depicted in Figure 4, where it is possible to visualize which pairwise mappings are affected by each filtering method.

The z-buffering filtering method can be formulated as:

(4) $$\begin{array}{cc} F^{\left(i\right)}=\; & \{f\in F:\neg \;f^{\left(i\right)}\cap g^{\left(i\right)}\;\vee \\ \; & \left(f^{\left(i\right)}\cap g^{\left(i\right)}\wedge \max_{v}\left(∥T_{i}\cdot x_{f,v}∥\right)<\min_{v}\left(∥T_{i}\cdot x_{g,v}∥\right)\right)\},\\ \; & \;\forall g\in F\;\backslash \;f,\;\forall v\in \{1,2,3\}, \end{array}$$

where $F^{\left(i\right)}$ is the set that contains the faces that are not occluded when considering the *i*-th image, $f^{\left(i\right)}$ and $g^{\left(i\right)}$ represent the projections of the *f*-th and *g*-th faces onto the *i*-th image, respectively, and $∥\cdot ∥$ denotes the $L_{2}$ norm. In summary, to assert whether the *f*-th face is occluded using the z-buffering filter, a comparison with respect to all other faces must be carried out. To conclude that the *f*-th face is not occluded by the *g*-th face, when considering the *i*-th image, one of two conditions must be met: the intersection between the projection of the vertices of the *f*-th face ($f^{\left(i\right)}$) and the projection of the vertices of the *g*-th face ($g^{\left(i\right)}$) must be the empty set; or, in case the intersection is different than the empty set, the maximum euclidean distance of the three vertices of the *f*-th, relative to the camera, must be less than the minimum euclidean distance of the three vertices of the *g*-th, face relative to the camera. Figure 4a,b contain the pairwise mappings between the same images shown in Figure 3, this time with the z-buffering filter applied. It is possible to observe that in Figure 4d the mappings on the right side of the boiler (zone limited by the black rectangle) were discarded because in Figure 4c they are occluded by the boiler itself. Note that the red colored mappings were not discarded by z-buffering, because this method can only discard pairwise mappings due to occlusions when both the occluded object and occluding object are represented in the 3D mesh. As detailed previously, we only have the 3D mesh of the corner of the room, meaning that the table does not exist in the 3D mesh. To tackle this variant of the occlusion, we propose the second filtering method-depth consistency. This method aims to discard occlusions due to discrepancies between the mesh and the point cloud. These discrepancies can have multiple sources, such as mesh irregularities, non-defined mesh, registration issues, among others. The main idea of depth consistency is to use information both from the 3D mesh and from the point clouds to estimate the depth of the faces via two different paths and then conclude, based on the difference of those values, whether depth inconsistency is present. Note that, by depth, we mean distance from the camera to the center of the face. When the depth values are inconsistent, we can assume that the face considered does not belong to the object that is being viewed by the camera. Therefore, using this mapping would introduce incorrect photometric mappings into the system. Our approach can be divided into two parts: (a) depth computation of the *f*-th face based on the 3D mesh; (b) depth computation of the *f*-th face based on the point cloud. The depth computation based on the 3D mesh is carried out by computing the $L_{2}$ norm of the vertices coordinates with respect to the camera, and then selecting the minimum value. To perform the depth computation based on the point cloud, we first use the partial point clouds from the setups where the images were taken and create depth images. Once the depth images are computed, the depth can be directly extracted using the coordinates obtained from vertex projection. The depth consistency filtering can be represented as:

(5) $$\begin{array}{cc} F^{\left(i\right)}=\{f\in F:\; & |\;\overset{\mathrm{mesh}}{\overset{︷}{\min_{v}\left(∥T_{i}\cdot x_{f,v}∥\right)}}-\overset{\mathrm{point}\;\mathrm{cloud}}{\overset{︷}{\min_{v}\left(D^{\left(i\right)}\left({\left[x_{f,v}^{\left(i\right)}\right]}_{y},{\left[x_{f,v}^{\left(i\right)}\right]}_{x}\right)\right)}}|<t_{dc}\}\;,\\ \; & \forall v\in \{1,2,3\}\;, \end{array}$$

where $F^{\left(i\right)}$ is the set that contains the selected faces using the depth consistency filter when considering the *i*-th image, $D^{\left(i\right)}$ corresponds to the depth image associated with the *i*-th image, and $t_{dc}$ is the depth consistency threshold, establishing the maximum admissible discrepancy between the distances computed using these two different methods.

Figure 4e,f contain the pairwise mappings with both the z-buffering filter and the depth consistency filter applied. By comparing them with Figure 4c,d, two differences should be highlighted: (a) the mappings inside the blue rectangle in Figure 4f were discarded because they are occluded by the left leg of the table in Figure 4e. Although one could expect those mappings to be removed with the z-buffering, that is not the case, since the leg of the table was not defined on the mesh, due to its reduced thickness, making z-buffering ineffective in this case. However, since the depth consistency filter compares the depth between the mesh and the point cloud, it was able to detect the discrepancy; (b) the mappings inside the brown rectangle in Figure 4f were discarded because the 3D mesh was not defined in that region, and as we can see, that is not a problem, since the depth consistency filter can detect and eliminate those false pairwise mappings.

Lastly, we propose a method to remove pairwise mappings in which the face has an excessively oblique viewpoint relative to the camera. We call this method camera viewpoint filtering. The assumption behind the removal of these pairwise mappings is that when the camera viewpoint is excessively oblique with respect to the face, the impact the registration error has on the accuracy of the pairwise mappings is amplified, potentially leading to the use of incorrect photometric information. Furthermore, this method allows us to discard mappings between the front and rear surfaces of a face, similarly to what is done in the back face culling method [51]. Let $\vec{f_{i}}$ be the unit vector with the direction from the *i*-th camera to the center of the *f*-th face, and $\vec{N_{f}}$ be the unit vector, normal to that face. The camera viewpoint filtering can be described as:

(6) $$\begin{array}{c} F^{\left(i\right)}=\left\{f\in F:\vec{f_{i}}\cdot \vec{N_{f}}<t_{cv}\right\}\;, \end{array}$$

where $F^{\left(i\right)}$ is the set that contains the faces that passed the camera viewpoint filter when considering the *i*-th image and $t_{cv}$ is a threshold that defines the admissible angle between $\vec{f_{i}}$ and $\vec{N_{f}}$.

Figure 4g,h contain the pairwise mappings with all filtering methods applied (z-buffering filtering, depth consistency filtering, and camera viewpoint filtering). Regarding the camera viewpoint filtering, the most significant difference is inside the zone marked with a black rectangle in Figure 4h, where the pairwise mappings that belong to faces that were being captured in an excessively oblique viewpoint were discarded.

In the present case study, this filtering procedure led to the removal of 74.23% of the pairwise mappings, which clearly demonstrates the very large amount of noise in this dataset. This reinforces the importance of using 3D information to discard incorrect pairwise mappings. Furthermore, this filtering procedure can have a huge impact in the image selection, because it allows us to avoid coloring a face with an image in which that face is occluded or with an excessively oblique viewpoint.

### 2.3. Joint Image Histogram Computation

**Figure 5:**
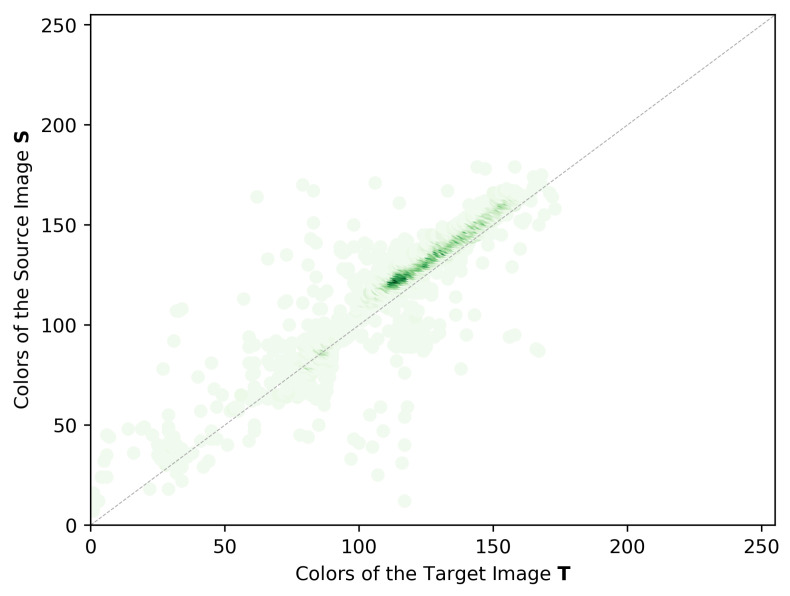
Example of the green channel of a JIH using the filtered pairwise mappings.

In this step, we compute a Joint Image Histogram (JIH) for each pair of images, using the filtered pairwise mappings. Let $x^{\left(i\right)}$, $y^{\left(k\right)}$ be the pixel coordinates of the mappings in the target image (**T**), and source image (**S**), respectively, and $I\left(x^{\left(i\right)}\right)$, $I\left(y^{\left(k\right)}\right)$ denote the the color intensity of the $x^{\left(i\right)}$ and $y^{\left(k\right)}$ pixel coordinates in the *i*-th image and *k-th* image, respectively. The JIH is built using the following equation:

(7) $$\mathrm{JIH}\left(x,y\right)=\sum_{\langle x^{\left(i\right)},y^{\left(k\right)}\rangle \in M}\delta \left(x,I\left(x^{\left(i\right)}\right)\right)\cdot \delta \left(y,I\left(y^{\left(k\right)}\right)\right)\;,$$

where δ(·) is the Kronecker delta function, and x and y represent all possible values of colors in **T** and **S**, being defined in the discrete interval $[0,2^{n}[$, where *n* is the bit depth of the images. Figure 5 shows an example of a JIH of the green channel. The green dots represent all the observations according to the mappings for that pair of images. A histogram is an observation count, which is represented by the color intensity of each point. The JIHs are used to estimate the CMFs, ${\hat{f}}_{m}$, which will then be used for producing the color corrected images.

### 2.4. Estimation of Color Mapping Functions

In this step, the current paper proposes to use the JIH as a base to estimate a Color Mapping Function (CMF), which are functions that map the colors of a source image **S** to a target image **T** resulting in a color corrected image $\hat{T}$. In this context, the CMF can be expressed as $\hat{x}={\hat{f}}_{m}\left(x\right)$, where $\hat{f_{m}}$ is the estimated CMF for each of the pairwise images, according to the mappings created, and $\hat{x}$ is the resultant color of the color corrected image $\hat{T}$ for a given color x of the target image **T**.

Since the CMF is a function, it cannot map a single element of its domain to multiple elements of its codomain. For the entire range of values (according to the bit depth of the image) for each color of the target image (x) there is one and only one resulting corrected value ($\hat{x}$). However, a typical JIH has several observations of y for each of the values in x, therefore it must be assumed that the JIH contains a set of considerably noisy observations. It is also important to note that, as there are three JIH for every pair of images, one for each channel, there are also three CMFs.

**Figure 6:**
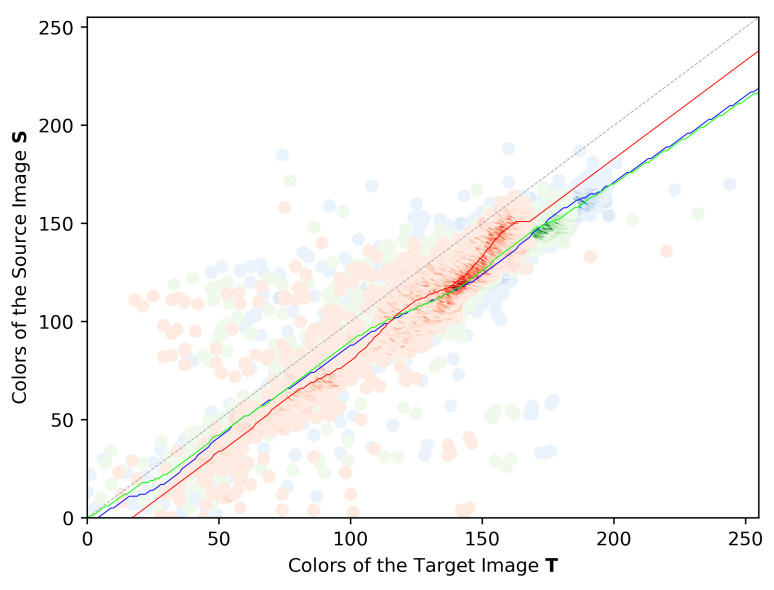
Example of the estimation of three Color Mapping Functions, one for each channel.

The current paper proposes to estimate the CMF using a regression analysis to fit the observations from the JIH. We utilize a regression analysis called Support Vector Regression (SVR) [52], making use of the Linear Kernel and the Radius Basis Function Kernel to estimate the single CMF approach used in the pipeline being presented. Figure 6 shows an example of the CMF estimation for a given JIH of a pair of images. The red, green and blue curves represent the CMFs estimation. For each channel, the function appears to fit the data of the JIH quite well, following the greater peaks of observations.

### 2.5. Pairwise Color Correction

In this step, we propose to perform what we call pairwise color correction by choosing a reference camera and using the CMF estimated previously for that given pair of images (**T**, **S**). The image from the reference camera will be the source image **S** and will form pairs with all the other images as target images **T**. The selection of the reference image by the user allows him/her to have control over the overall appearance of the color corrected mesh. The user may select a darker image because that is his preference, and with that as reference the algorithm will color correct all images to appear similar to the reference one, resulting in a darker appearance textured mesh. Another user may have a different opinion, select another image as reference, which would guide the algorithm to produce a textured mesh with a different appearance. For these reasons, we view this arbitrary selection of the reference image as an advantage rather than a shortcoming of the proposed approach.

Note that it would also be possible to automatically select a reference image. One criteria is to select the image which has a wider range of colors, so that the reference contains as many color examples as possible. Another could be the selection of the image that contains more overlap with other images, to ensure that there are sufficient color mappings between the reference image and the others, thus ensuring that the estimation of the color mapping function is based on a large number of color mapping samples.

Even though the CMF was estimated from a JIH created using only the filtered pairwise mappings, the current paper proposes the generalization of the CMF to color correct all pixels of the target image **T**. This generalization increases the similarity between all the images, not only within the mappings, but in the entire image, showing that our color correction approach can handle multiple images.

For each pair of images there are three color mapping functions that were estimated previously, one for each channel. That means that each of the three channels will be color corrected independently. To perform the color correction, the color value x of each pixel of the target image **T** will be given as input to the CMF and then replaced by the color corrected value $\hat{x}$. After performing the color correction for each channel independently, the channels are merged to generate the corrected image $\hat{T}$.

**Figure 7:**
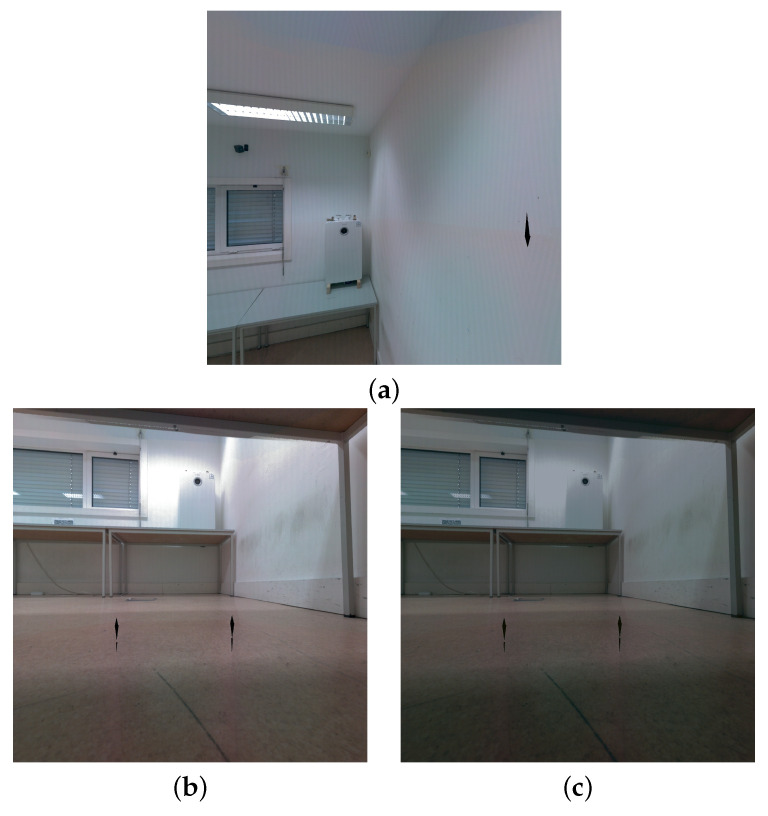
Example of the color correction for a pair of images: (**a**) Chosen reference camera (source image **S**); (**b**) Original target image **T**; (**c**) Color corrected target image $\hat{T}$.

**Figure 8:**
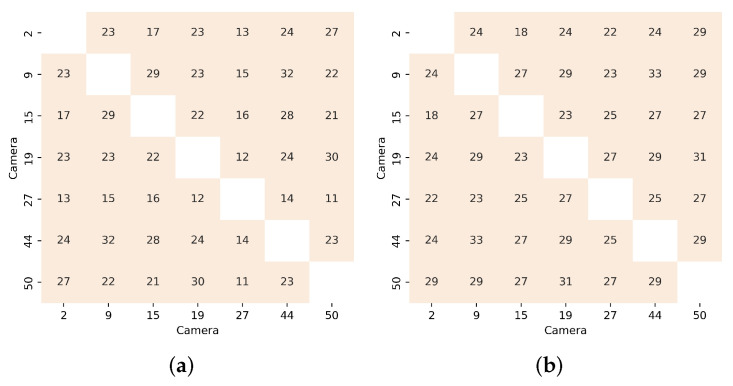
Confusion Matrices using PSNR metric which the higher the score, the more similar the pair of images are: (**a**) Original images (no color correction); (**b**) Corrected images with proposed approach.

Figure 7 shows an example for a pair of images. As we can observe, the target image **T** became more similar to the source image **S**, indicating that the color correction was successful. Figure 8 shows the effectiveness of the proposed approach to handle the color correction of multiple images and increase their similarity. The objective of the current paper is to evaluate the quality of the color correction by examining, not only the similarity of the images with respect to each other, but by analyzing the success of their use in the improvement of the textured mesh quality.

### 2.6. Image Selection

The last step to produce the textured mesh is what we refer to as image selection. The goal is to select, for each face, the image used to colorize that face. This process of colorization is based on cropping, from the selected image, the projection of the face. We propose to use, not only the corrected images to create a higher-quality mesh, but also the information associated with the filtering of pairwise mappings (Section 2.2). To select the image to colorize the *f*-th face, in addition to the information provided by the filtering pairwise mappings, we need a criteria to select one image from the available images. We implemented two different criteria: random selection and selection based on the largest area of the projection. The random approach selects a random image from the available ones. To analyze the impact of the color correction in the textured mesh, this is the best approach because it amplifies the perception of the dissimilarities between all images. The selection based on the largest area of the projection consists of selecting the image where the area of the face projection is larger. This method produces higher-quality textured meshes, but it is less suitable to evaluate the impact of the color correction because it produces meshes with less image transitions between adjacent faces.

**Figure 9:**
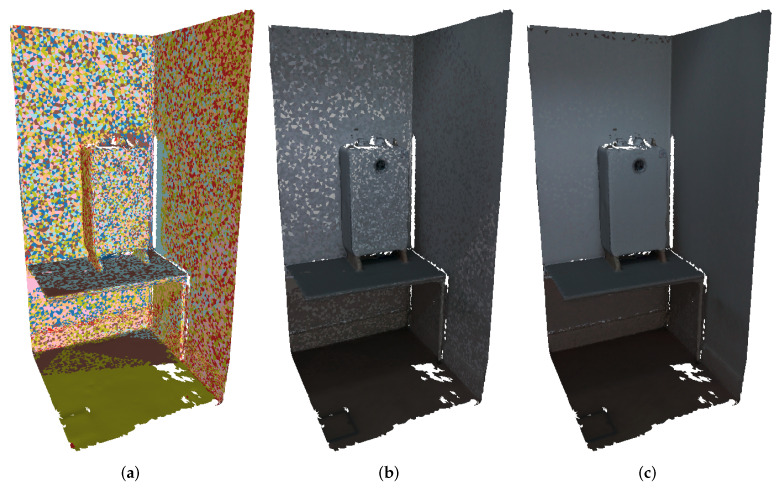

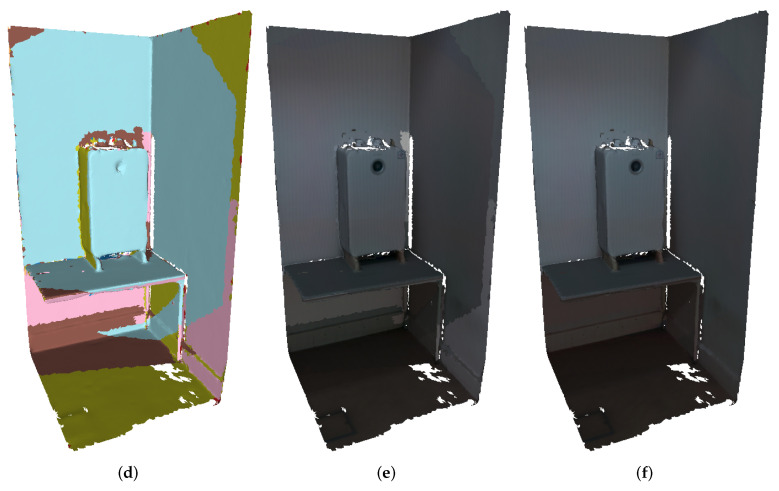
Textured meshes produced. (**a**,**d**) illustrate the result of the random selection and the selection based on the largest area of the projection, respectively. The color of each face is associated with the selected image, meaning that faces with the same color have the same selected image. The resultant textured mesh with random selection as the image selection technique is depicted in (**b**) using the original images, and in (**c**) using the color corrected images. The resultant textured mesh with image selection based on the largest area of the projection is depicted in (**e**) using the original images, and in (**f**) using the color corrected images.

Figure 9a,d showcase the result of image selection using the random selection and largest projection area selection, respectively. In Figure 9a it is possible to observe that the random selection appears to be a more suitable image selection technique to assess the effectiveness of the color correction procedure, since it uses several images to colorize the faces within the same surfaces, leading to the creation of multiple texture seams artifacts. While the largest projection area selection tries to use the same image to colorize all faces on a certain surface, the number of zones where a transition of the selected image exists is reduced, decreasing the likelihood of texture seams artifacts, and with it, the impact of color correction. Figure 9b,c represent the textured mesh produced with the random selection technique, using the original and corrected images, respectively. The positive impact of the color corrected images on the textured mesh is considerable, eliminating most of the visual unappealing artifacts, and thus creating a more visually appealing textured mesh. Figure 9e,f contain the same structure as Figure 9b,c, but showcase the image selection based on the largest area of the projection. We can observe that the quality of the textured mesh using the color corrected images has improved, but since the number of transitions between images is smaller, the improvement is less noticeable. Nonetheless, the overall appearance of the textured mesh is highly affected by the smoothness of transition between images, and as we can see in Figure 9, our proposed color correction approach produces smoother transitions, leading to higher-quality textured meshes.

## 3. Results

Section 2 presented some preliminary results to illustrate each of the stages of the pipeline; this section contains the complete results, showcasing the color corrected images and their impact on the visual quality of the meshes from different viewpoints. Firstly, a comparison between the proposed approach and other state-of-the-art color correction algorithms is presented. This was divided into three sections, in order to analyze images and textured meshes. Section 3.1 and Section 3.2 present image-based quantitative and qualitative evaluations, respectively. Section 3.3 presents mesh-based qualitative evaluations. In Section 3.4 and Section 3.5 we discuss the impact of the proposed filtering methods, and of the selection of the reference image, on the quality of the color correction process.

Table 1 lists all the evaluated algorithms. Algorithm #1 is the baseline approach, which means that no color correction is performed. Algorithm #2 is a global approach proposed in [24], which uses a linear transformation to impose the global color distribution of a source image onto a target image, in the lαβ color space. Algorithm #3 is an adaptation of Algorithm #2, in which only the lightness channel of the lαβ color space is corrected (luminance correction). Algorithm #4 is the approach proposed in [33], which uses a root-polynomial regression to estimate a CMF. Algorithm #5 uses the Vandermonde matrix to compute the coefficients of a polynomial [42] that is applied as a CMF. In both #4 and #5, the unfiltered pairwise mappings are used to produce the color correspondence vectors required by these approaches.

Section 2.5 presented the pairwise color correction, where a reference image is selected as source to form pairs with the remaining images as target images. Since the dataset contains seven images, six pairs of images are formed to be directly color corrected, and we refer to those pairs of images as Subset 1. Additionally, in order to analyze the capacity of the approaches to increase the color similarity between all the images, a Subset 2 is formed, composed by image pairs that were not directly color corrected, meaning neither of the images was the source in the color correction procedure. Subset 2 is therefore composed of 15 pairs of images, obtained from the combination of the available images, excluding the reference image. These two subsets combined form the complete dataset, which contains 21 pairs of images. These groups of images will be used for the quantitative evaluations.

The quantitative performance evaluation of each color correction algorithm is determined employing two different image similarity metrics over the filtered pairwise mappings. The usage of the metrics solely with the filtered pairwise mappings is the best way to fairly compare the robustness of each algorithm because it allows us to analyze the overlapping portions of each pair of images without being influenced by the abundance of noise present before filtering. The first metric, proposed in [23], is known as peak signal-to-noise ratio (PSNR). It is a similarity measure, so the higher the score values, the more similar the pair of images are. The second metric is named CIEDE2000. It is a dissimilarity metric, meaning that more similar images result in lower score values. This metric is adopted as the most recent color difference metric from the International Commission on Illumination (CIE), and was improved in [53].

### 3.1. Image-Based Qualitative Evaluation

**Figure 10:**
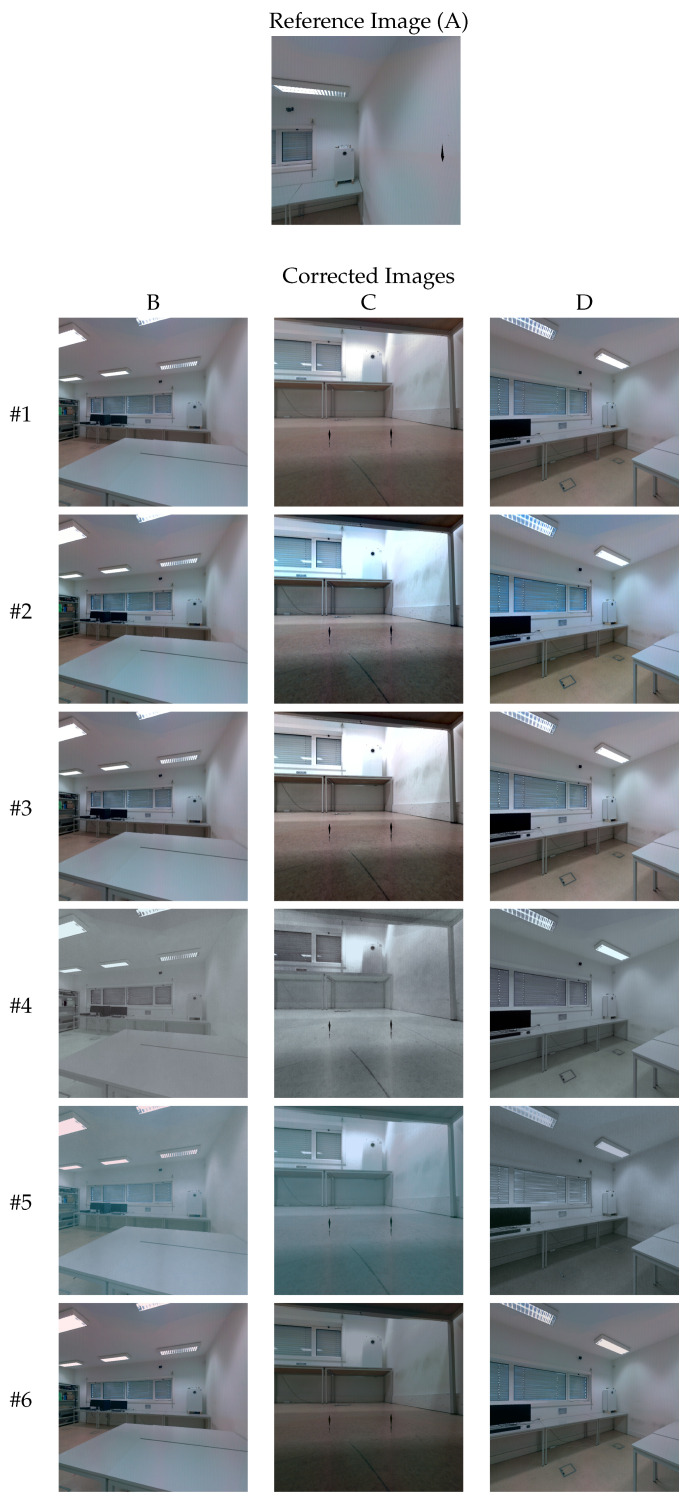
Corrected images (**B**–**D**) (columns) using image (**A**) as reference image (source image). Each row corresponds to a different color correction algorithm.

A comparison between the approaches (Table 1) is presented by qualitative evaluation of the color similarity between images. As described in Section 2.5, the pairwise color correction is performed in 6 pairs of images (Subset 1) that were created with the same reference image. After the procedure, the target images should have become more similar in color to the reference image (image A in this case) and, as a consequence, more similar with respect to each other as well. Figure 10 shows the color corrected images produced by each approach. For clarity reasons, we randomly choose three of the six images available. In the first row (algorithm #1), the target images B, C, and D are shown in their initial form (uncorrected). Afterwards, global approaches #2 and #3 seem to have excessively increased the brightness of the images. Between the two, #3 appears to have performed a marginally better color correction. For algorithm #4, the corrected images have become more similar to each other. However, the overall color palette obtained appears quite different from the reference image. The images in #5 are quite degenerated, more noticeably images B and C. From the analysis of the obtained results, the most similar images in color to the reference image were produced by the proposed approach (#6). In image C (algorithm #6), the reduction of brightness is evident on the left side of the boiler, which improved the color similarity with both the reference image and the other images. It is noteworthy that, even though the pairwise mappings were created only for the boiler zone, the generalization of the CMFs in the other parts of the image achieved very good color correction results as well.

### 3.2. Image-Based Quantitative Evaluation

In this section, an evaluation of the similarity between images using the PSNR and CIEDE2000 quantitative metrics is presented. Table 2 shows the results separately for the following sets: Subset 1, Subset 2, and the complete dataset. Global approaches #2 and #3 could not deal with the distinct color clusters in order to compute a good global color distribution, even with the slight improvement obtained through the adaptation made in algorithm #3. Algorithms #4 and #5 were the second and third best approaches, respectively, considering both image similarity metrics (PSNR and CIEDE2000) in all sets. The proposed approach outperformed all the other algorithms, in all sets, and in both metrics. Concerning the PSNR similarity metric, the proposed approach achieved averages scores of 27.50, 26.60, and 26.86 in Subset 1, Subset 2 and Dataset, respectively. For the CIEDE2000 dissimilarity metric, the proposed approach achieved average scores of 3.48, 4.09, and 3.91 in Subset 1, Subset 2 and Dataset, respectively.

As expected, Subset 1 achieved the best overall scores because it contains only pairs of images that were directly color corrected. It is worth noting that Subset 2 also achieved very good scores, confirming the capacity of the proposed approach to increase the color similarity between multiple images. The only algorithm besides the proposed approach that achieved better overall results than the baseline approach was algorithm #4. These results reinforce the notion that, to accomplish a successful color correction, it is not only important to estimate better CMFs, with high-level techniques, but also to effectively reduce the amount of noise in the input data, caused by registration errors, occluded surfaces, incorrect photometric correspondences, among others.

### 3.3. Mesh-Based Qualitative Evaluation

**Figure 11:**
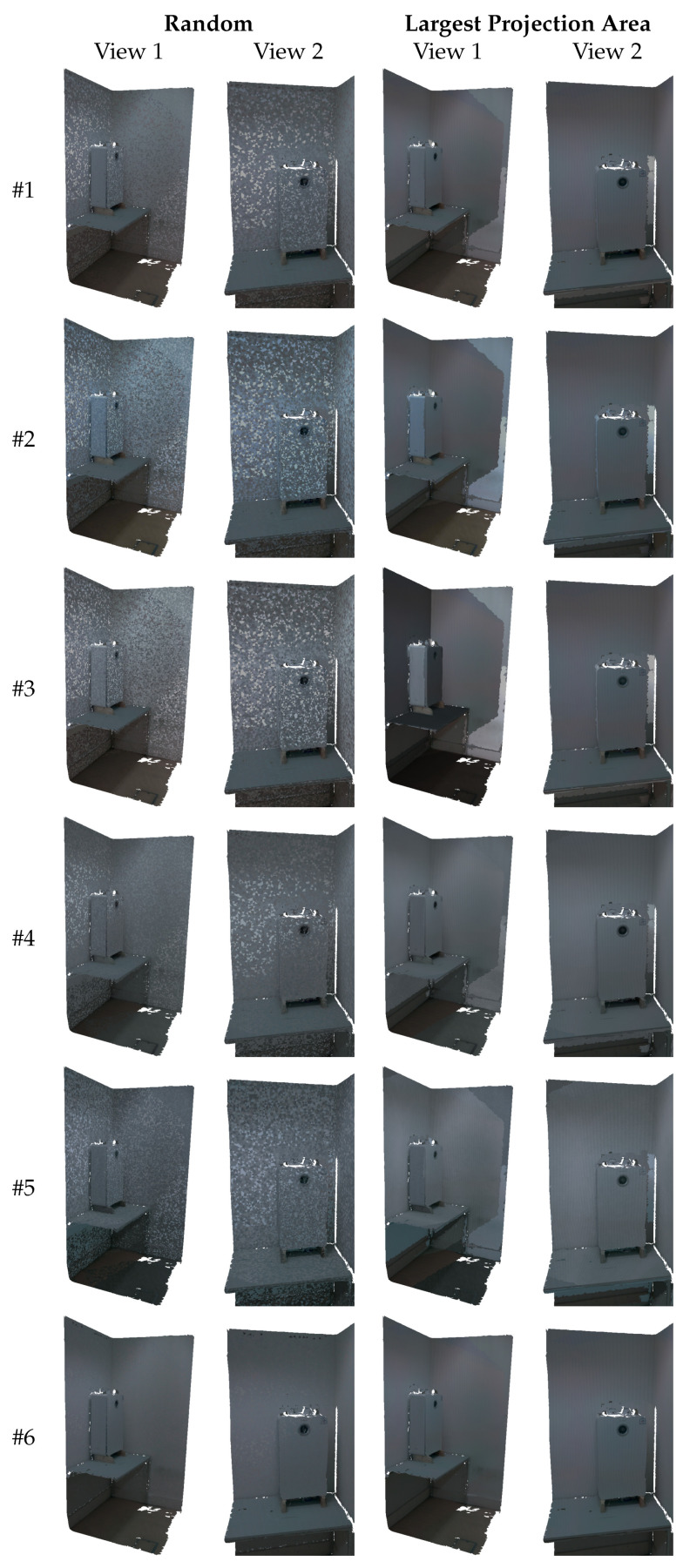
Textured meshes produced by each approach from two different viewpoints and using two different image selection techniques: random selection and largest projection area selection.

In this section, a comparison between the approaches (Table 1) is presented by evaluating the visual quality of textured meshes created using two different image selection criteria: random selection and largest projection area selection. In order to achieve a fair evaluation, all the meshes produced by each approach have exactly the same texture mapping for both images selection techniques. As discussed in Section 2.6, the usage of multiple images to texture a mesh will result in the transition between images to map different areas of the mesh. The apparent color difference at the limits between neighboring triangles creates visual artifacts, known as texture seams. Figure 9a,d illustrate where these transitions are present in the mesh, using representative colors. Figure 11 presents the textured meshes for each approach, using the two image selection techniques, in two different viewpoints. The first viewpoint shows an overview of the entire mesh. The second viewpoint shows a zoomed-in region of the boiler and the wall.

Since algorithm #1 uses the original images, the visual artifacts created by the changes in the selected image are noticeable. The random image selection criterion aggravates the effect of this problem, but it can be mitigated by color correcting the images. The meshes produced by the approaches #2 through #5 appear unable to improve the visual quality of the textured mesh, presenting a large amount of very noticeable texture seams. The proposed approach (algorithm #6) appears to produce the highest quality textured mesh.

Even when using the random image selection criterion, the texture seams are less noticeable, verifying that indeed the images become more similar in color with respect to each other. Moreover, with the largest projection area selection criterion, the visual quality of the mesh raises even more, displaying almost no texture seams. These results demonstrate the robustness of the proposed approach by showing a significant improvement in the visual quality of the textured mesh.

### 3.4. Impact of the Proposed 3D-Based Filtering Methods

In this section, the objective is to evaluate the impact each filtering method has on the color correction outcome. For that, we evaluate four variants of the proposed approach, listed in Table 3.

**Figure 12:**
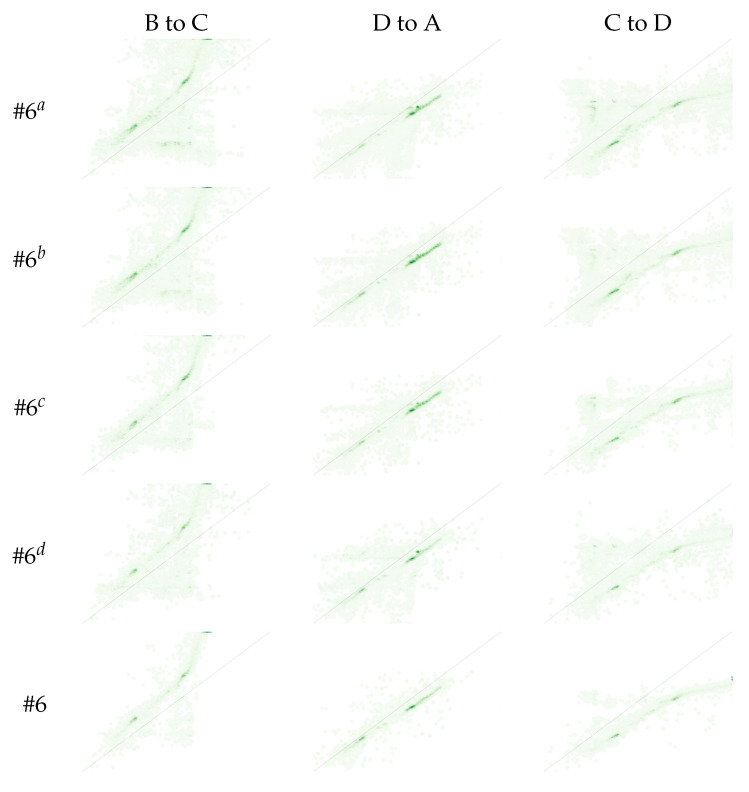
JIHs produced using the different variants of the proposed approach (rows). Each column represents the green channel of the JIH between a randomly chosen pair of images. For example, the first column, B to C, represents the green channel of the JIH between image B and image C.

We propose to evaluate the impact of each filtering method from two perspectives: assessing the percentage of pairwise mappings removed by analyzing the JIH (Table 4 and Figure 12); using image similarity metrics (Table 5).

Table 4 depicts the average percentage of pairwise mappings removed when considering all available image pairs (Dataset). Algorithm #6 removed 74.23% of the pairwise mappings, demonstrating the importance of using robust techniques to handle noise. Through an individual analysis of each filtering method, we can note that algorithm $\#6^{d}$ was the component that resulted in the largest reduction of the pairwise mappings. On the other hand, algorithm $\#6^{b}$, was the one with the least impact on the pairwise mapping removal. Figure 12 illustrates the impact that each approach has on the JIH. As was expected, due to the 74.23% reduction of the pairwise mappings, algorithm #6 completely transforms the JIH when compared to the JIH produced using the unfiltered pairwise mappings (algorithm $\#6^{a}$). The JIHs produced by algorithm #6 contain less correspondences of colors in the source image for each color in the target image, which is exactly what we expect from a noise free JIH. In other words, algorithm #6 is able to produce thinner JIHs, which enable the estimation of more accurate CMFs, leading to higher levels of color similarity between images. The JIH depicted in cell (#6, D to A) is a very good approximation of a noiseless JIH. Based on the conclusions extracted from Table 4, it was expected that the individual filtering approach that should have the most impact on the transformation of the JIH was algorithm $\#6^{d}$, which is not the case. The reason for this is that the majority of the pairwise mappings removed by algorithm $\#6^{d}$ belong to zones where the JIH is denser, and therefore the difference is not highly noticeable. This is an interesting conclusion because it shows that to evaluate the effectiveness of a filtering method, in addition to assessing the number of mappings removed, an analysis of the zones of the JIH where those mappings are must be carried out. The individual filtering approach that produced thinner JIHs was algorithm $\#6^{c}$. Although in this dataset, the most significant filter was algorithm $\#6^{c}$, it is important to note that the effectiveness of each filter depends on the characteristics of the dataset used.

Table 5 shows the quantitative results achieved by all algorithms in the same sets as in Table 2: Subset 1, Subset 2, and Dataset. As expected, the pairs of images in Subset 1 present a higher level of color similarity than the pairs of images in Subset 2. For the Subset 1, algorithm #6 achieved the best results both in the PSNR metric and CIEDE2000 metric, with average scores of 27.50 and 3.48, respectively. For the Subset 2, when considering the PSNR metric, algorithm $\#6^{b}$ achieved an average score of 26.86, followed by algorithm #6−26.60. Regarding the CIEDE2000 metric, algorithm #6 was, once again, the approach that achieved the best results, with 4.09. Finally, when considering the entire dataset, algorithm $\#6^{b}$ achieved an average score of 27.00 in the PSNR metric, and in the CIEDE2000, the best results belong to algorithm #6, with an average score of 3.91. CIEDE2000 takes into account the sensibility of human-eye to certain colors [53], making this image similarity metric more suitable for image-based comparisons, and therefore, we consider that algorithm #6 was the approach that achieved the best results. It is noteworthy that both algorithm $\#6^{b}$ and algorithm $\#6^{c}$ in all sets achieved remarkable results, being quite close to the score achieved by algorithm #6.

From these results, we can conclude that the usage of the 3D-based filtering methods is a significant contribution to the quality of the color correction procedure.

### 3.5. Impact of the Selection of Reference Image on Image-Based Quantitative Evaluation

One of the hyperparameters that must be defined in the proposed approach is the selection of the reference image from all available images. The goal of this section is to evaluate the impact that this selection has on the color correction outcome. Table 6 contains the quantitative results achieved when selecting each image as the reference. Note that, although the reference image is different, all other configurations are the same. For Subset 1, image G achieved the best average score concerning the PSNR metric (29.00), while in the CIEDE2000 metric, the reference image that attained the best average score was image D, with a value of 3.38. Regarding Subset 2, the reference image that achieved the best average score when considering the PSNR metric was image B (27.40), and concerning the CIEDE2000 metric, image A achieved an average score of 4.09. Finally, regarding the entire Dataset, image A achieved the best results in both the PSNR metric and CIEDE2000 metric, with average scores of 26.86 and 3.91, respectively. These results demonstrate that the effectiveness of the color correction is not strongly dependent on the selection of the reference image. The results using image C as the reference image achieved the worst performance in both evaluation metrics when considering all sets. It is noteworthy that, using this image, the results for the PSNR metric in Subset 1 were subpar to those obtained in Subset 2. This can be explained by the fact that image C was taken under a table, see Figure 10, which caused a different illumination from the rest of the images, hampering the color correction process. Still, algorithm #6 using image C as the reference still outperforms algorithms #2, #3 and #5, achieving equivalent results to algorithm #4, see Table 2. Any other image used as reference for the proposed approach produces results that outperform the other evaluated algorithms.

## 4. Conclusions

This paper proposed two main contributions to the state-of-the-art of color correction: a pairwise-based methodology capable of color correcting multiple images from the same scene; the usage of 3D information to filter out noisy mappings between pairs of images. The pairwise-based methodology consists of selecting one image as the reference image and then color correcting all other images with respect to the reference image. This procedure increases, not only the color similarities of all images with respect to the reference image, but also the color similarities between all image in the dataset. The usage of 3D information consists of applying 3D information from the real world, namely point clouds and meshes, to build a filtering procedure capable of producing more reliable spatial registration between images, which results in more accurate color correction.

Results demonstrate that the proposed approach outperforms all other approaches, both using qualitative and quantitative metrics. Furthermore, results show that our color correction procedure enhances the visual quality of the produced textured meshes. We tested two different image selection criteria, random selection and largest area projection selection, and demonstrated that even using the most simple one-random selection, the proposed approach produces textured meshes virtually free of texture seams. In addition, we explored how each filtering method affects both the JIH and the color correction outcome, where we demonstrated that all filtering methods play an important role in the color correction procedure. Using the filtering tools that we propose, it is also possible to quantify the amount of noise that exists in datasets used for color correction, which in our case was around 75%. This large value shows clearly that robust techniques are very important to achieve a successful color correction procedure. In the end, we performed a comparative analysis, where we show that the effectiveness of our proposed approach is not strongly dependent on the selected reference image.

For future work, we plan to explore several techniques to estimate color mapping functions in order to take full advantage of the filtered pairwise mappings that we obtain using the filtering procedure proposed in this paper. In addition, we intend to integrate other color correction algorithms with our filtering procedure and evaluate the impact that our filtering procedure has on the color correction outcome. We also plan to test our color correction procedure in scenarios with a wider color range, in order to analyze its robustness concerning different scenarios.

## Figures and Tables

**Table 1:** Algorithms compared with the proposed approach.

Algorithm #	Reference	Description
#1	-	Baseline (target image)
#2	Reinhard et al. [24]	Global Color Transfer
#3	Reinhard et al. adapted [24]	Global Color Transfer—Lightness Channel
#4	Finlayson et al. [33]	Root-Polynomial Regression Color Correction
#5	De Marchi et al. [42]	Vandermonde Matrices for Color Correction
#6	This paper	3D-based Color Correction

**Table 2:** Mean and Standard Deviations of the PSNR and CIEDE2000 (CIED) scores for each algorithm in Subset 1, Subset 2, and Dataset. Best results highlighted in bold.

	Subset 1				Subset 2				Dataset			
	PSNR		CIED		PSNR		CIED		PSNR		CIED	
Alg.	μ	σ	μ	σ	μ	σ	μ	σ	μ	σ	μ	σ
#1	22.33	5.31	8.42	4.90	21.00	6.20	9.11	5.30	21.38	6.00	8.91	5.19
#2	19.05	5.60	11.10	6.63	16.39	5.09	14.17	6.68	17.15	5.38	13.29	6.81
#3	19.00	5.69	10.83	6.26	16.49	5.31	13.24	6.72	17.21	5.54	12.55	6.68
#4	23.00	4.83	6.97	3.85	21.47	5.31	7.50	3.83	21.90	5.23	7.35	3.84
#5	20.83	3.72	8.83	4.37	19.07	5.54	11.07	6.12	19.57	5.15	10.43	5.77
#6	**27.50**	5.92	**3.48**	0.39	**26.60**	4.08	**4.09**	1.21	**26.86**	3.71	**3.91**	1.08

**Table 3:** Variants of the proposed approach used to evaluate the impact of the filtering methods.

Algorithm	Description
$\#6^{a}$	Proposed approach without any filtering method
$\#6^{b}$	Proposed approach using only z-buffering
$\#6^{c}$	Proposed approach using only depth consistency
$\#6^{d}$	Proposed approach using only camera viewpoint
#6	Proposed approach

**Table 4:** Average percentage of pairwise mappings removed by each algorithm.

Alg.	μ	σ
$\#6^{a}$	0.00	0.00
$\#6^{b}$	26.27	13.06
$\#6^{c}$	39.71	7.45
$\#6^{d}$	56.41	15.17
#6	74.23	10.81

**Table 5:** Mean and standard deviation of the PSNR and CIEDE2000 (CIED) scores for each algorithm. Best results highlighted in bold.

	Subset 1				Subset 2				Dataset			
	PSNR		CIED		PSNR		CIED		PSNR		CIED	
Alg.	μ	σ	μ	σ	μ	σ	μ	σ	μ	σ	μ	σ
$\#6^{a}$	26.50	2.99	4.27	0.77	26.26	3.97	4.80	1.47	26.33	3.72	4.65	1.33
$\#6^{b}$	27.33	2.49	3.83	0.46	**26.86**	3.70	4.28	1.28	**27.00**	3.41	4.16	1.13
$\#6^{c}$	27.00	2.77	3.95	0.53	26.53	4.06	4.35	1.41	26.67	3.75	4.24	1.24
$\#6^{d}$	26.33	3.14	4.22	1.03	26.20	4.02	4.93	1.54	26.24	3.79	4.72	1.45
#6	**27.50**	5.92	**3.48**	0.39	26.60	4.08	**4.09**	1.21	26.86	3.71	**3.91**	1.08

**Table 6:** Mean and standard deviation of the PSNR, CIEDE2000 (CIED) scores for each reference image selection, using algorithm #6. Best results highlighted in bold.

	Subset 1				Subset 2				Dataset			
	PSNR		CIED		PSNR		CIED		PSNR		CIED	
Ref.	μ	σ	μ	σ	μ	σ	μ	σ	μ	σ	μ	σ
A	27.50	5.92	3.48	0.39	26.60	4.08	**4.09**	1.21	**26.86**	3.71	**3.91**	1.08
B	23.50	3.25	4.55	1.73	**27.40**	2.65	4.19	0.81	26.29	3.34	4.29	1.16
C	21.33	1.97	6.28	0.86	22.27	3.53	7.96	2.76	22.00	3.19	7.48	2.49
D	28.00	3.21	**3.38**	0.66	25.07	3.66	4.47	1.40	25.90	3.78	4.19	1.36
E	27.67	3.68	3.48	0.78	25.20	3.58	4.60	1.54	25.90	3.78	4.28	1.46
F	25.33	4.50	4.55	2.06	25.33	3.81	4.91	1.29	25.33	4.02	4.81	1.55
G	**29.00**	1.15	4.00	0.55	25.67	3.94	5.20	1.98	26.62	3.71	4.86	1.78

## Data Availability

Not applicable.

## Abbreviations

The following abbreviations are used in this manuscript:

BTF	Brightness Transfer Function
CMF	Color Mapping Function
EM	Expectation Maximization
GMMs	Gaussian Mixture Models
CIE	International Commission on Illumination
JIH	Joint Image Histogram
PSNR	Peak Signal-to-Noise Ratio
**S**	Source Image
S-CIELAB	Spatial CIELAB
SVR	Support Vector Regressor
**T**	Target Image
$\hat{T}$	Corrected Image
